# Preparing autistic workers in work adjustment: Employer and employee perspective analysis

**DOI:** 10.4102/ajod.v15i0.1794

**Published:** 2026-03-11

**Authors:** May L. Elfina

**Affiliations:** 1 Department of Psychology, University of Muhammadiyah Malang, Malang, Indonesia

**Keywords:** disability, workers, work adjustment, wellbeing, autism worker

## Abstract

**Background:**

The value of diversity in human resources, particularly empowering employees with disabilities as assets, has been investigated by industry. Even in an era of economic expansion, the existence of workers with disabilities, particularly those with autism, is stigmatised.

**Objectives:**

This study aimed to understand how employees with autism spectrum disorder (ASD) adjust their work and how their employers view their performance.

**Method:**

Mixed method design is the method used in this study. By purposive sampling, three employees with ASD were chosen as the main subjects, and their degree of job adjustment was assessed using the Work Adjustment Illinois Scale (WAIS). Three employers were also interviewed in depth to get qualitative data. Validity, reliability and normality checks were all part of quantitative analysis. Work Adjustment Illinois Scale scores are also categorised using a statistical method (mean ± standard deviation) before qualitative analysis is performed using the interactive analysis method.

**Results:**

The results show that the majority of workers with ASD exhibited moderate to high degrees of work adjustment. Self-confidence, the necessity of structural modification, and the significance of employer comprehension were among the themes that surfaced from the processing of the qualitative data.

**Conclusion:**

Workers with ASD are able to make good work adjustments when facilitated with work adjustments from both the individual themselves and support from their workplace.

**Contribution:**

In order to support the continued professional success of people with ASD, the study highlights the necessity of inclusive work rules and training for supervisors and coworkers to support the wellbeing of workers with autism.

## Introduction

Because of the Disability Law’s implementation, businesses are under pressure to hire 1% of their workforce from private enterprises and 2% of their workforce from the government (Republic of Indonesia 2016). The rule sets a standard for fair employment opportunities for people with impairments, particularly in the formal sector. About 20% of individuals with disabilities are employed in the formal sector, while the remaining 80% are employed in the unorganised sector (Maghfirah 2022). Even though employers are still only partially implementing the aforementioned rule, a number of big corporations have started to show concern for the equality of people with disabilities. As demonstrated by the Indonesian experience, different stakeholders must work together to develop and formulate policies pertaining to the inclusion of workers with impairments (Syahfitri et al. 2024). According to the National Autistic Society (NAS), a British research agency, only 16% of the 2000 adult autistic adults hold part-time jobs, despite the fact that 77% of them said they desired to work. Without comprehensive understanding, individuals with autism will be highly protected, miss out on schooling, and undoubtedly have a harder time obtaining employment (NAS 2016). Because of high out-of-pocket costs and unemployment, caregivers of children with autism spectrum disorder (ASD) frequently face severe financial crises; they also frequently deal with a variety of psychosocial issues, including stress, depression, anxiety, worsening physical health and strained marital relationships (Tathgur & Kang 2021). Their ability to cope will be impacted by extreme stress and worry. Asking for assistance can be challenging for autistic workers, which might make it harder for them to adapt to their positions (Hayward et al. 2023).There are many inclusive strategies that companies can attempt including autism awareness training, low-stimulus (non-complex) work, clear instructions and flexible working hours (Petty et al. 2023b). Employers must recognise the qualities that autistic employees offer to the workplace, such as attention to detail, honesty and strong focus on task in other way, clear job description and detailed instruction should be provided in the workplace. However, employers need to understand the needs of their employees and provide workplace conditions that allow autistic workers to do their jobs well (Equality and Human Rights Commission 2010).

Research with coverage of autistic workers is very limited, especially in Indonesia, so ongoing research is needed as a concern for autistic workers. This study was conducted as a scientific basis to determine the description of the work environment for people with disabilities, especially autism, namely how the perception of the work situation needed by autism, both from the perspective of employers and autistic workers themselves. The data that will be gathered are an in-depth description of the pattern of physical access and workplace, sensory environment, social environment and communication and also the condition of work adjustment on the autistic workers. The problem-solving approach in this study is to conduct an in-depth study through mixed methods so that an effective and efficient concept can be concluded about the preparation of autistic workers. Global prevalence increased by 1.9% and incidence by 5.20% between 1990 and 2021, according to the Global Burden Disease report (Tathgur & Kang 2021). The autistic population is increasing; however, adequate employment opportunities have not kept pace with their capabilities. In addition, the phenomenon of work adjustment for autistic workers in companies experiences quite high obstacles. This study examines the environmental conditions preferred by autistic workers and aims to provide an in-depth understanding to help employers design work environments that align with autistic workers’ perspectives, ultimately promoting work adjustment and work-related wellbeing.

Work rehabilitation can be understood as a process through which employees regulate the fit between themselves and their work environment, commonly referred to as work adjustment (Strauser et al. 2021). The study found that self-efficacy mediates the link between person-organisation fit and employee work adjustment, enabling employees to adapt more effectively when they perceive a good organisational fit (Tathgur & Kang 2021). Self-efficacy is a crucial theory to support the application of motivation in individuals so that it can improve better work results (Bandura 1997). This can be explained by a person’s ability to participate in a vocationally driven manner, which will affect their perception in facing the demands of their work. Strengthening autism awareness training to increase understanding of arrangements that may be made in the workplace is vital, according to several prior studies undertaken to explain the phenomenon of autistic workers; nevertheless, only a small proportion of employees have finished the training (Petty et al. 2023b). Workplace modifications such as flexible hours, clear written instructions, sensory-friendly lighting, and quieter environments should be implemented in a balanced manner to avoid creating unfair disadvantages or safety concerns for other staff, while enhancing the well-being and productivity of autistic employees (Petty et al. 2023b). The results of this study emphasise the need for a more compassionate and inclusive workplace that genuinely enables autistic workers to flourish, as well as the need for employers to be more proactive in recognising and adopting workplace modifications (Davies et al. 2022). In order to promote career self-efficacy in people with special needs, it is also critical to create a career plan for them (Elfina & Andriany 2023).

The purpose of this study is to determine the perspective of autistic workers in carrying out their work in terms of work adjustment theory in order to achieve employee wellbeing. This study provides a new perspective on how the theory can be used to explain the work adjustment process carried out by workers with autism in meeting job demands, while meeting their personal needs. In addition, this study also contributes to the development of the concept of employee wellbeing, especially by highlighting the role of work adjustment in creating a balance between individual needs and organisational expectations. Life, work and psychological requirements in both work and life are the three fundamental components of employee wellbeing; it is not only how employees feel and perceive their level of life and job satisfaction but also their psychological experiences and the degree of contentment they demonstrate in both areas (Zheng et al. 2015). Workplace wellbeing is correlated with inclusion (Randel 2025). This perspective is an important addition to the literature that supports diversity and inclusion in the workplace. In practice, the results of this study can be a guide for employers in understanding the special needs of workers with autism and strategies that can be used to create a supportive work environment. The information generated can help companies to design relevant training and mentoring programmes for developing both workers’ technical and social skills. In addition, this study can be a basis for formulating more inclusive work policies, including the provision of certain facilities or flexibility that support the productivity and wellbeing of workers with autism. Furthermore, this study is expected to increase public and organisational awareness of the importance of supporting workers with special needs, so that a more friendly, inclusive and diversity-supporting work environment is created.

## Research methods and design

### Design and study setting

The research method approach used in this study is mixed-method study. This study employs a mixed-methods design with an exploratory sequential approach. The first phase uses a qualitative method through in-depth interviews as the primary data source to explore participants’ experiences and meanings. The second phase applies a quantitative method using psychological scales to strengthen and map the qualitative findings. Data integration is conducted at the interpretation stage, where the quantitative results are analysed and explained based on themes derived from the qualitative analysis. All autistic workers and employers who employ autistic people in various regions can participate in this study according to the results of the sampling process carried out by the researcher. The research subjects were determined through purposive sampling. The criteria for subjects in this study are autistic workers who are working full-time or part-time with a target of three subjects. Furthermore, significant others, people who significantly influence a subject’s life and assist in moulding their objectives, routines and self-perception are known as significant others. In this case, the researcher used the employers as significant others for each subject, and they will be added to each subject. So that the total subjects in this study are six people.

The stages and procedures of the research were carried out systematically with a literature review as the preparation stage, implementation with qualitative methods, and an in-depth data analysis process to achieve research outputs. The research flowchart starts from the literature review stage, instrument preparation, collection of interview data and survey data, data analysis stage including data reduction, data presentation, drawing conclusions, and the final report stage that was Analysis of Employer and Worker Perspectives: Preparing Autistic Workers as a Foundation for the Formation of Work Adjustment to Achieve Employee Well-Being.

### Instruments

We used the interview as the primary data in this research, so semi-structured interviews have been used for the subjects. We prepared two interview guidelines: (1) for the ASD workers; and (2) for the employers. As the supplementary data, we used questionnaire (Work Adjustment Illinois Scale [WAIS]) to measure the work adjustment of each ASD workers to complete the qualitative data. Before conducting quantitative data analysis as secondary data and qualitative data analysis as primary data, the researcher conducted several classical assumption tests on quantitative data acquisition. Among them, the instrument reliability value, calculated using Cronbach’s alpha, was 0.677, indicating moderate and acceptable internal consistency. To test the data distribution, a Shapiro–Wilk normality test was conducted on the combined WAIS scores of the three subjects. The results showed that the data were not normally distributed (*W* = 0.274, *p* < 0.001), so a non-parametric analysis approach was more appropriate. The instrument that is used in this research is Illinois Work Adjustment Scale (Strauser et al. 2021). By this instrument we can also categorise the range of work adjustment level using hypothetic categorisation. By hypothetic categorisation we found that, if the score is X ≥ 124 it means a high work adjustment, if the score ranged 114 < X < 124 it means moderate work adjustment, and if the score is X ≤ 114 it means a low work adjustment.

### Data analysis

This study used two data analysis techniques: Quantitative analysis that used quantitative descriptive and qualitative data analysis that used the interactive analysis model from Miles and Huberman (Sugiyono 2016), namely: (1) data reduction (data collection) namely summarising, choosing the main things, focusing on important things, looking for themes and patterns; (2) data presentation (data display) with narrative text with the aim of making it easier to understand the data obtained; and (3) the process of verification or drawing conclusions results in new findings or conceptual descriptions of phenomena, which may take the form of causal or interactive explanations, such as hypotheses or theories.

### Ethical considerations

This study was approved by the Research Ethics Commission of the Faculty of Psychology, Universitas Muhammadiyah Malang, Indonesia (Approval No. E.6m/241/KE-FPsi-UMM/XII/2025). The study adhered to fundamental ethical principles, including beneficence, non-maleficence, and respect for participants. All participants provided informed consent prior to data collection. Participation was voluntary, and participants were informed of their right to withdraw at any time without consequence. Confidentiality and anonymity were strictly maintained throughout the research process.

## Results

### Demographic

This study was conducted on three workers diagnosed with ASD by professionals and the three workers had quite different backgrounds such as job, last education, and experience of work can be seen in Table 1.

**TABLE 1:** Sociodemographic characteristics of the study participants.

Aspect	Subject 1	Subject 2	Subject 3
Initial Name	AN	KM	AZ
Sex	Female	Male	Male
Domicile	Singapore	Indonesia	Indonesia
Ethnic	Javanese, Dayak	Javanese	Javanese
Job	Editor	Admin and finance	Cleaning service
Work experience (in years)	10	2	2
Age by onset ASD diagnosed (in years)	33	2	2
ASD severity (diagnosed by professional)	Level 2	Level 2	Unknown
Last education	Bachelor’s degree	Bachelor’s degree	Vocational High school

ASD, autism spectrum disorder.

### Quantitative analysis

#### Work adjustment categorisation of autism spectrum disorder workers

Table 2 presents the categorisation of work adjustment among workers with ASD using the Illinois Work Adjustment Scale. The results show that two subjects have work adjustment in the moderate category, and one subject shows work adjustment in the high category.

**TABLE 2:** Work adjustment categorisation of autism spectrum disorder workers.

Subject	Work adjustment categorisation
AN	Moderate
KM	High
AZ	Moderate

#### Results of descriptive analysis of reasonable adjustments for autism spectrum disorder workers

This criterion is based on the reasonable adjustment by Petty et al. (2023b). The demographic characteristics of the participants are presented in Table 1. The implementation of reasonable adjustments is presented in Table 3, which summarises the application of adjustment criteria in the workplaces of employees with autism spectrum disorder (ASD). The instrument consists of two sections: Checklist A and Checklist B. Both sections contain identical items representing various workplace adjustments designed to support employees with ASD. Checklist A assesses adjustments that have been previously observed or implemented, including clear work guidelines, autism-related training, visual or verbal instructions, structured schedules, regular feedback, reassurance during stressful situations, modifications to the physical environment, autism-awareness training for co-workers, explanations of policy changes, recruitment adjustments, and flexible working arrangements. Checklist B evaluates the current availability or potential feasibility of these same adjustments within the workplace. Together, the two sections allow comparison between implemented practices and future readiness for inclusive employment.

**TABLE 3:** Implementation of adjustment criteria in autism spectrum disorder workplace.

Answer	AN	KM	AZ	Total
Yes	7	12	11	30
No	6	1	2	9

As shown in Table 3, a total of 30 ‘Yes’ responses and 9 ‘No’ responses were recorded across the three subjects (AN, KM, and AZ). A ‘Yes’ response indicates that the adjustment criteria have been implemented, whereas a ‘No’ response indicates non-implementation. KM reported the highest number of implemented adjustments (12), followed by AZ (11) and AN (7). In contrast, AN reported the highest number of non-implemented adjustments (6), compared to AZ (2) and KM (1). These findings suggest variation in the level of workplace adjustment implementation across participants.

### Qualitative analysis

#### Results of qualitative analysis of autism spectrum disorder workers through presentation of result themes

The analysis of participants’ experiences revealed seven core themes: self-awareness of ASD, recruitment facilitated by family and social networks, job and environmental suitability, general rather than ASD-specific training, interests and activities outside of work, sensory and social challenges, and supportive office structure and regulations. These interconnected themes provide a nuanced understanding of how individuals with ASD navigate and sustain employment within various workplace settings.

Participants demonstrated a strong sense of self-awareness regarding their ASD diagnosis, often developing this understanding during adolescence. One participant, AN, recalled suspecting her condition at the age of 12 years old, because of feeling different from her peers, but got a formal diagnosis when she was 33 years old. Similarly, AZ reported receiving a formal diagnosis during junior high school, while KM became aware of characteristic symptoms before entering elementary school, and his parents gave him interventions to cope with these conditions. This early awareness often informed their coping strategies and helped to shape their personal and professional trajectories. AN recognised that she has an ASD lately, but she could feel something different with her:

‘12 years old already suspected, because I felt different from my friends.’ (AN)

The recruitment process was largely shaped by informal support systems, particularly family members and social connections. KM credited her mother’s friend for facilitating her employment opportunity, whereas AN entered the workforce through an internship secured via university networks. These accounts highlight the reliance on personal relationships rather than formal mechanisms in gaining access to employment, emphasising the critical role of social capital for individuals with ASD:

‘My mother was referred by her friend, to hire me as a worker at that time.’ (KM)

In terms of job fit and environmental compatibility, all participants reported a high level of comfort and perceived support in their current roles. AN described her job as ‘very comfortable’ and found its positive impact on productivity. AZ, who had worked in several settings, particularly appreciated a workplace located close to home for its convenience and flexibility. These reflections underscore the importance of matching job roles and workplace environments to individual needs in order to foster optimal performance and wellbeing. AN said that she gets a huge flexibility from the employer, and she realises it makes her more productive:

‘This job is very comfortable, it has an effect on productivity.’ (AN)

Training experiences reported by the participants were generally not tailored to the needs of individuals with ASD. According to KM’s employer, all employees received standardised training. Meanwhile, in a different workplace setting, AZ engaged in self-directed learning and was also provided with additional intensive training by his employer to enhance his performance. In addition, KM got a very supportive employer, to give a broad chance to develop his competencies, and promise of referral to a better company, so that KM can develop his knowledge and practice. AN said that she did not get any training from her employer, but that it is not a big problem, because of being an editor her job is highly flexible and she can afford everything well. Not every company could give an appropriate adjustment. This indicates a lack of inclusive training practices that consider neurodiversity and suggests a gap in organisational efforts to prepare ASD individuals for workplace integration.

Beyond the workplace, participants showed a wide range of interests and pursued meaningful activities in their personal time as their passion. It means that every subject has their own preferred activity that shows their hobbies reflecting their personal passion or aptitude. AN, for instance, expressed a passion for writing and shared that she had authored two books while staying at home. Other participants engaged in learning programming or attending professional training programmes, reflecting both creative capacity and a strong intrinsic motivation to continue learning and growing outside formal employment structures.

Despite their successes, some participants experienced sensory and social challenges in the workplace. KM reported that overstimulating environments, such as crowded spaces, could lead to stress. However, he emphasised that with appropriate support and adjustments, these difficulties did not interfere significantly with her ability to work. These accounts point to the importance of sensory-aware workplace design and social understanding in promoting sustainable employment for neurodivergent individuals. Even about the uniform that she had to use, AN said that she felt uncomfortable using uniform because of the kind of thread of the uniform.

Lastly, participants described their workplaces as generally supportive, especially regarding structure and regulations. Furthermore, AN emphasised that flexible rules were ‘very helpful’, while KM highlighted the importance of tolerance in scheduling as a form of indirect but meaningful accommodation. These organisational practices, although not always explicitly designed for ASD support, proved effective in fostering a more inclusive and accommodating work environment.

In conclusion, the findings suggest that individuals with ASD benefit greatly from early self-awareness, informal recruitment networks, appropriate job placements and flexible organisational structures. While most workplace training remains generalised, personal initiative and strong external interests often compensate for the lack of ASD-specific support. Importantly, when sensory and social needs are acknowledged and addressed through practical accommodations, individuals with ASD are able to thrive and maintain productive, fulfilling work performance.

#### A summary of qualitative data analysis results on autism spectrum disorder workers

- **Entry paths to the work are highly dependent on family and social networks:** No formal inclusive recruitment process was found. Access to work is dominated by informal channels such as references from parents or acquaintances. A more open and inclusive recruitment system is needed so that people with ASD do not have to rely solely on personal networks.
- **Informal work environment adaptation has a big impact:**
- Although there is no specific training or rules for ASD workers, the flexibility and tolerance provided informally provide high comfort and better productivity. Small interventions such as flexible working hours, working from home, and co-workers’ understanding of sensory needs can significantly improve performance:
- **Autism spectrum disord er individuals demonstrate high creative capacity and dedication:** Many workers with ASD develop additional skills independently, such as writing books or learning programming, reflecting self-directed learning and strong perseverance. These findings suggest that career development programs and advanced training may be particularly beneficial when designed flexibly and aligned with individual interests.
- **Key challenges:** Sensory and social interaction, not job competence. Difficulties are more likely to arise in the form of sensory overstimulation or social communication demands, rather than technical incompetence. Workspace designs and work rules that are more friendly to neurodivergent needs need to be developed in companies. For instance, desk positions were strategically arranged to reduce direct exposure to bright lighting and high noise levels, in line with the sensory characteristics of autistic employees. Furthermore, adjustments were made to the uniform fabric, as certain types of materials caused sensory discomfort, which in turn interfered with the employees’ focus and work performance.

## Discussion

The results of the study showed that the level of work adjustment in workers with ASD in this study ranged from moderate to high, with one subject showing a high score and two others moderate. This suggests variability in cognitive functioning and daily living skills among individuals with ASD, with workplace conditions and social support playing a significant role in shaping these abilities. This finding is in line with a study by Lorenz et al., which found that work achievement and job stability in individuals with ASD are greatly influenced by the level of fit between personal characteristics and work environment conditions (Lorenz et al. 2016). This is comparable to the work adjustment theory, which holds that employee productivity can be maximised by a combination of environmental factors and personal traits. According to the job adjustment theory, employees’ self-adjustment is a result of their surroundings and themselves strengthening one another until they are satisfied (Rogelberg 2017).

Qualitative analysis of interviews with ASD workers revealed three main themes, presented in Table 4: Confidence in competence; social adaptation strategies; and the need for clear work structures. Autism spectrum disorder workers who were in the high work adjustment score category tended to demonstrate confidence in carrying out tasks, stated that they felt capable of working independently, and understood instructions well. This supports the results of a study by Costley et al. (Costley & Baldwin 2014), which stated that high cognitive abilities in ASD workers were positively correlated with their success in completing work tasks independently and efficiently.

**TABLE 4:** A summary of themes from the results of data analysis of disability workers.

Theme	Sub-theme	AN	AZ	KM
Confidence in competence	Self-awareness about ASD	Understand that she was different for a long time	Not explicitly mentioned about diagnosis	Understood from early life, and accepted
	Work suitability	Very suitable, feel comfortable	Feel suitable in graphic design field, but now he gets a job as cleaning service	Comfortable working with clear structure and tasks
	Interest and personal activity preference	Likes writing, solitary activities	Enjoys making animations and music	Enjoys playing games
Social adaptation strategies	Recruitment track	The company hired her through LinkedIn	Through friendship networks	Own initiative, with family support
	Support from work environment	Given her own workspace, tasks do not change	Superiors and colleagues understand that he needs time alone	Given a clear work structure and not many changes
	Challenges in sensory and social interaction	Difficulty interpreting colleagues’ social expressions	Confusion and discomfort in crowded environments	Experiences social pressure at work and occasional meltdowns when exposed to overwhelming sensory stimuli from coworkers
Need for clear work structure	Obstacles faced	Sometimes misinterprets people’s expressions	Difficulty in verbal communication, and cannot understand a complex information	Quickly anxious, difficulty initiating communication
	Training or support from employer	Attending non-specific ASD training	Self-taught, no specific training	Received assistance when starting work

ASD, autism spectrum disorder.

Meanwhile, workers with moderate work adjustment scores tend to rely more on clear work structures and supervision. They expressed the need for routine and difficulty in dealing with sudden changes. This finding is supported by the study carried out by Macdonald et al. (2018), which highlighted that structuring tasks with task lists or numbered instructions, providing clear visual cues about and what to do next is very important for ASD.

From the employer’s perspective, several key areas were identified, including understanding ASD characteristics, implementing flexible work policies, and providing training for management and coworkers. Employers of workers with high scores emphasised more on autonomy and work efficiency, while employers of workers with moderate scores focused more on the need for supervision and communication adjustments. This supports the research of Hedley et al. (2016). While organisations involved in the NAS Prospects programme were more likely to recognise the importance of a supportive environment for individual development, those without prior experience employing individuals with ASD tended to emphasise skill level.

Furthermore, employers who have had positive experiences in employing individuals with ASD emphasise that ASD workers have advantages in focus, thoroughness, and commitment to work. This view is supported by the study of Scott et al. (2015), which states that despite challenges in social communication, ASD workers often demonstrate strengths in aspects of work that require attention to detail and consistency. However, challenges remain, particularly because of line managers’ lack of understanding of disability inclusion. Many managers and human resources professionals report feeling unprepared to accommodate diverse types of disabilities (Praslova 2023). This lack of understanding can widen the gap between the cognitive potential of ASD workers and their realised productivity in the workplace.

In general, the results of this study indicate an observed relationship between work adjustment categories and adaptation strategies, support needs and employers’ perceptions of the performance of workers with ASD. Although not explicitly intended, this finding emerged consistently across interviews and was therefore included in the analysis. To establish an inclusive workplace for individuals with impairments, awareness and acceptance campaigns are required (Rachmawati et al. 2022).

The results of this study indicate that workers with ASD are self-aware of their condition and are able to access the world of work through the help of family or social networks. This informal route is their main strategy because there is no established inclusive recruitment system in Indonesia. This is in line with the report of *The Jakarta Post*, which states that individuals with autism often rely on social support because of minimal access to an inclusive formal work system, even a cubicle room to reduce their stress (Belinda 2022). Those realising the support, family is a key factor in mediating the transition process to the world of work.

Regarding the work environment, ASD workers in this study felt comfortable when they received a clear work structure, consistent tasks, and a less crowded work environment. Informal adjustments were proven to be effective in supporting their performance. It is crucial to hire autistic employment consultants and educate providers and staff on autism (Hayward et al. 2023). Adaptation does not always have to be in the form of sophisticated technology or big policies, but can be in the form of interpersonal understanding and flexible work arrangements. However, challenges still arise, especially in terms of sensory and social interactions. Several participants complained of difficulty in understanding social expressions, speaking in a crowded environment or dealing with social pressure at work. This reflects the general characteristics of ASD, as explained by Bury et al. (2020), where the main challenges for ASD individuals are social challenges as prominent phenomena and a risk factor for people with autism in the workplace.

Another interesting aspect of this result is how employers view employees with ASD. All three employers stated that they rated the performance of workers with ASD positively, especially in terms of accuracy, discipline and perseverance. They did not see significant challenges during the work process; however, most of them did not make formal adjustments. Employers of KM and AZ had a good knowledge of ASD, especially the employers of AZ. He has a child with ASD, so he knows very well how to interact with and give a chance to a person with ASD. Interview data revealed that both employers developed their knowledge of ASD due to personal concern and proactive learning. They also suggested that organisational awareness of ASD is still insufficient in many workplace settings. This is consistent with a study by Teresa, Basto & Cepellos (2023), which showed that in many companies, although managers’ judgements are predicated on stereotypes, they ultimately evolve as a result of their interactions with professionals who have ASD.

In the context of policy, Indonesia has *Law No. 8 of 2016* concerning persons with disabilities, which guarantees the right to work and be treated fairly. However, the implementation of this policy still has a gap, such as the unequal understanding of the concept of inclusion and the low readiness of companies to adapt (Putri & Nugraha 2024). Because of this, people with ASD continue to rely mostly on their personal connections or good fortune when looking for work. This study highlights that, with the right assistance and accommodations, employees with ASD have a lot of potential. In addition to being competent workers, these employees also contribute value through their tenacity, integrity and reliability. Applying the work inclusion concept will eventually assist not only people with ASD but also the organisation’s work culture by bringing in a variety of viewpoints and methods of operation, such as the unique capacity of people with ASD to perform repetitive activities, identify patterns and possess specialised competencies (Krzeminska et al. 2019). The study’s recommendations include training for employers, interventions and the creation of particular work adjustment tools for employees with ASD. Longitudinal research is recommended to assess the long-term development and impact of partnerships with public and private organisations in promoting inclusive employment for individuals with ASD.

## Conclusion

Based on the results of a qualitative analysis of the experiences of workers with ASD and the perspectives of employers, it can be concluded that individuals with ASD have good work potential if they receive appropriate support from the work environment. The dominant path to work through family support shows that there are still more limitations in the formal inclusive recruitment system than in informal work adjustments. From the employer’s perspective, there is a positive perception of workers with ASD, but there is still minimal implementation of structurally inclusive work adjustments or policies. However, the three employers showed quite good acceptance and personal support. This is an important basis for increasing awareness and education about the importance of creating a sustainable inclusive work environment. Based on these findings, it is recommended that the government, educational institutions and the business world work together to provide training and work assistance systems for individuals with ASD.
